# Effects of a pre-enlargement on fracture incidence of reused reciprocating instruments: A clinical study

**DOI:** 10.1590/0103-644020246147

**Published:** 2024-12-16

**Authors:** Gustavo De-Deus, Guilherme Alves Palhares, Emmanuel João Nogueira Leal da Silva, Felipe Gonçalves Belladonna, Diogo da Silva Oliveira, Kilber Duarte Carvalho, Erick Miranda Souza, Marco Aurélio Versiani

**Affiliations:** 1 Department of Endodontics, Fluminense Federal University, Niterói, Rio de Janeiro, Brazil; 2 Department of Endodontics, State University of Rio de Janeiro, Rio de Janeiro, Brazil; 3 Department of Dentistry II, Federal University of Maranhão, São Luís, Maranhão, Brazil; 4 Dental Specialty Center, Brazilian Military Police, Minas Gerais, Brazil

**Keywords:** Instrument fracture, molar, pre-enlargement, reciprocating movement, root canal preparation

## Abstract

This study assessed the impact of pre-enlarging the root canal using a size 25 K-file on the fracture incidence of three reciprocating instruments after multiple uses. After a glide path performed with a size 15 K-file, the instruments (Reciproc R25, Reciproc Blue R25, and WaveOne Gold Primary) were initially used in 936 root canals. Then, for the second and third uses (933 and 927, respectively), a pre-enlargement using a size 25 K-file at the working length was done. Periapical radiographs assessed fragment location and length, and chi-square tests compared observed and expected frequencies (α = 5%). The fracture incidence during reuse ranged from 0% to 0.64%. Reciproc did not fracture during reuse (*P* = 0.99), but experienced two fractures in the third use (*P* = 0.000). Reciproc Blue and WaveOne Gold each had one fracture during the second (*P* = 0.000) and third (*P* = 0.000) uses. Following a pre-enlargement using a size 25 K-file for the second and third uses in 1,860 canals, only 6 instruments fractured (0.32%), suggesting that this approach may prolong the lifespan of these instruments and serve as an alternative for their reuse with a reduced risk of fracture.

## Resumo

Este estudo avaliou o impacto do pré-alargamento do canal usando uma lima K de tamanho #25 na incidência de fraturas de três instrumentos reciprocantes após múltiplos usos. Após um *glide path* realizado com uma lima K de tamanho #15, os instrumentos (Reciproc R25, Reciproc Blue R25, e WaveOne Gold Primary) foram inicialmente usados em 936 canais radiculares. Depois, para o segundo e terceiros usos (933 e 927 canais, respectivamente), um pré-alargamento usando uma lima K de tamanho #25 no comprimento de trabalho foi realizado. Radiografias periapicais avaliaram a localização e o comprimento dos fragmentos, e os testes qui-quadrado compararam as frequências observadas e esperadas (α = 5%). A incidência de fraturas durante o reuso variou de 0% a 0,64%. Reciproc não fraturou durante o reuso (*P* = 0,99), mas sofreu duas fraturas no terceiro uso (*P* = 0,000). Reciproc Blue e WaveOne Gold tiveram, cada um, uma fratura durante o segundo (*P* = 0,000) e terceiro (*P* = 0,000) usos. Após o pré-alargamento do canal usando uma lima K de tamanho #25 em 1.860 canais, apenas 6 instrumentos fraturaram (0,32%), sugerindo que esta abordagem pode prolongar a vida útil desses instrumentos e servir como uma alternativa para sua reutilização com risco reduzido de fratura.

## Introduction

Nickel-titanium (NiTi) instruments improve root canal shaping by enhancing efficiency, precision, and patient comfort compared to stainless-steel manual preparation; however, their susceptibility to deformations and fractures entails a potential risk of treatment failures ^1^. To mitigate this risk, the pre-enlargement of the root canal space using small hand files has been recommended. This process usually comprises three key steps: scouting, apical patency, and glide path. Canal scouting involves the initial negotiation of the canal using passive, small, and flexible files to progress toward the apex up to the provisional working length (WL) ^2^. Apical patency focuses on passing a small file beyond the root length to ensure complete access to the main apical foramen. Following the determination of the WL, a glide path is established to create a smooth and reproducible trajectory from the main canal orifice to the foramen, typically achieved when a stainless-steel size 10 or 15 K-file fits loosely in the canal ^3^. These preliminary procedures aim to extend the lifespan of mechanical instruments used in subsequent canal enlargement by effectively managing torsional stress, thereby decreasing the likelihood of iatrogenic mishaps ^4^.

Manufacturers of NiTi instruments have also expressed concern about the issue of instrument fractures, addressing it through strategies such as modifying design, developing thermally treated NiTi alloys, and employing different kinematics like reciprocating motion ^5^ ^,^ ^6^. Reciprocating motion has been shown to improve instrument lifespan ^7^ by relieving stress and increasing fatigue resistance ^7^ ^,^ ^8^; furthermore, it mitigates the risk of torsion fractures, allowing the safe shaping of root canals using a single instrument, even without the creation of a glide path ^9^. This method, however, subjects the instrument to significant mechanical stress ^10^, prompting manufacturers to advise its single use. For instance, Reciproc M-Wire (VDW GmbH, Munich, Germany), Reciproc Blue (VDW GmbH), and WaveOne Gold (Dentsply Sirona, Ballaigues, Switzerland) instruments are specifically designed as "forced" single-use instruments, featuring a heat-sensitive plastic ring to prevent reuse. Despite this established single-use policy, the growing support for reusing reciprocating instruments in both clinical and laboratory investigations ^11^ ^,^ ^12^ has sparked an ongoing debate in the literature on this topic.

In a systematic review and meta-regression study, the critical clinical factor influencing the risk of NiTi instrument fractures during root canal treatment was identified as the number of uses ^13^. The study noted that while some research, including studies conducted by undergraduate students ^14^ ^,^ ^15^ ^,^ ^16^, reported decreased instrument fracture rates ^9^ ^,^ ^11^ ^,^ ^15^ ^,^ ^16^ ^,^ ^17^ ^,^ ^18^, there were inconsistencies in studies with similar methodologies ^15^ ^,^ ^16^ ^,^ ^17^, underscoring the need for further investigation. Reusing NiTi instruments entails a heightened risk of fractures due to torsional stress, which occurs when the instrument's tip becomes lodged in the root canal walls ^19^. So, in theory, pre-enlarging the root canal to a size similar to the instrument's tip would prevent its entanglement in the canal walls, thereby minimizing the likelihood of fractures induced by torsional stress. Therefore, this study sought to assess how the pre-enlargement of the root canal using a size 25 K-file influences the incidence of fractures of 3 reciprocating systems (Reciproc, Reciproc Blue, and WaveOne Gold) after 2 or 3 uses. The null hypothesis tested was that there would be no significant differences regarding the incidence of fractures among the tested systems.

## Materials and Methods

This clinical study, approved by the local ethics committee (Protocol 39455720.3.0000.5243) adhered to ethical principles following the World Medical Association Declaration of Helsinki. Informed consent was obtained from all patients, and the guidelines of the STROBE Statement ^20^ have been adopted. To ensure strict criteria for sample inclusion, a small effect size of 0.1 was used. With an alpha error of 0.05 and a power beta of 0.95, the results indicated that a minimum total size of 1,545 root canals (approximately 515 molar teeth with 3 canals) was necessary to detect significant differences in the comparisons (G*Power 3.1; Heinrich Heine, Universität Düsseldorf, Germany).

### Sample selection and groups

The study sample consisted in 932 maxillary and mandibular molars from 918 patients (14-80 years-old) (Tables 1 and 2) who underwent root canal treatment from April 1, 2021, to April 1, 2022 at a private clinical practice in Rio de Janeiro, Brazil. A preoperative periapical radiograph was taken (digital sensor 5100; Carestream Dental, Atlanta, GA, USA) confirming that all selected teeth had fully formed root apices, visible canals, no previous endodontic treatment, no resorption, no fracture, and canal curvatures of less than 20 degrees. Digital radiographs in a buccolingual direction were taken to calculate the angle of the curvature using AxioVision 4.5 software (Carl Zeiss Vision GmbH, Hallbergmoos, Germany), according to Schneider’s method ^21^.

Each tooth had at least 3 canals, but extra canals such as the second mesiobuccal canal of maxillary molars, middle mesial canals of mandibular molars, and canals encased in extra roots (*radix*) were excluded. Thus, the study involved the treatment of 2,796 root canals using Reciproc R25 (size 25/.08v), Reciproc Blue R25 (size 25/.08v), or WaveOne Gold Primary (size 25/.07v), with each group using a total of 104 instruments.

**Table 1:** Number of maxillary and mandibular molars (n = 932) that underwent root canal treatment with Reciproc (RC), Reciproc Blue (RB) and WaveOne Gold (WG) instruments (n = 104 per group) after 1, 2 or 3 uses.

	Maxillary			Mandibular				
	1^st^ molar	2^nd^ molar	3^rd^ molar	1^st^ molar	2^nd^ molar	3^rd^ molar	Teeth	Canals
RC (1 use)	33	25	-	25	17	4	104	312
RC (2 uses)	33	19	-	30	20	2	104	312
RC (3 uses)	27	21	1	27	23	5	104	312
RB (1 use)	23	22	1	24	28	6	104	312
RB (2 uses)	27	13	1	33	27	3	104	312
RB (3 uses)	31	20	1	24	21	6	103	309
WG (1 use)	31	20	2	22	25	4	104	312
WG (2 uses)	37	16	1	26	20	3	103	309
WG (3 uses)	23	10	2	36	26	5	102	306
Total	265	166	9	247	207	38	932	2796

**Table 2:** Demographic data (gender and age) of 918 patients who underwent root canal treatment with Reciproc (RC), Reciproc Blue (RB), and WaveOne Gold (WG) instruments after 1, 2, or 3 uses.

	Gender		Age						
	Male	Female	10-20	21-30	31-40	41-50	51-60	61-70	71-80
RC (1 use)	38	65	7	18	12	12	20	19	15
RC (2 uses)	37	65	11	17	15	12	17	16	14
RC (3 uses)	41	61	15	12	15	12	13	17	18
RB (1 use)	38	65	9	21	9	22	17	11	14
RB (2 uses)	39	64	17	19	10	17	21	12	7
RB (3 uses)	38	63	7	14	18	10	18	16	18
WG (1 use)	39	64	13	16	12	14	14	18	16
WG (2 uses)	45	54	16	13	17	12	20	12	9
WG (3 uses)	40	62	8	16	12	16	9	25	16
Total	355	563	103	146	120	127	149	146	127

### Experimental procedures

The first use of each instrument was performed according to the following protocol. After conventional access cavity preparation, patency was verified with a size 10 K-file (Dentsply Sirona) and the WL determined at the main foramen using an apex locator (VDW Gold; VDW GmbH). Glide path was done with a size 15 K-file (Dentsply Sirona) and then, root canal shaping was performed with instruments adapted to a 6:1 angle handpiece (VDW Gold; VDW GmbH). Reciproc R25 and Reciproc Blue R25 instruments were utilized in “RECIPROC ALL” mode, and WaveOne Gold Primary instruments were employed in “WAVEONE ALL” mode, with the assignment of each system to specific teeth being randomly determined (www.random.org). The preparation involved a slow in-and-out pecking motion with ⁓3 mm amplitude and light apical pressure, where the instrument was withdrawn and cleaned after each set of 3 pecking motions, repeating the process until the WL was attained. Irrigation was performed with a total of 15 mL of 2.5% NaOCl per canal, followed by a final rinse with 5 mL of 17% EDTA for 3 minutes and 5 mL of distilled water using a syringe fitted with a 30-G NaviTip needle (Ultradent, South Jordan, UT, USA) positioned 2 mm from the WL.

After the chemomechanical preparation, a periapical radiograph was taken to determine the presence of instrument fragments in the root canal. In the event of instrument separation, the patient was properly informed, and efforts were made to remove or bypass the separated instrument. The root canals were dried using absorbent paper points according to the root canal system used, and then filled using the single cone technique, in which a gutta-percha cone (Reciproc R25, Reciproc Blue R25, or WaveOne Gold Primary) coated with the AH Plus sealer (AH Plus sealer; Dentsply Sirona) was slowly inserted into the canal to its WL. The excess of gutta-percha was trimmed off with an electrical heat carrier 1 mm below the orifice and vertically compacted with a cold plugger. After that, the access cavity was restored with resin composite (Filtek Z350 XT; 3M ESPE, St. Paul, MN, USA). Each instrument was examined under ×8 magnification (OPMI Pico; Carl Zeiss, Oberkochen, Germany) for major defects or deformations, and if any plastic deformation was identified, the instrument was promptly discarded. No instrument was discarded. In the absence of alterations, the heat-expanded ring was carefully removed using a sterile scalpel blade before the instrument underwent cleaning in an ultrasonic bath with an enzymatic detergent for 20 minutes, followed by thorough drying. Each cleaned instrument was then packaged individually and sterilized through autoclaving at 132 °C for 30 min. The preparation procedure during the second and third use of the same instruments mirrored the protocol for the first use, except for the pre-enlargement using a size 25 K-file (Dentsply Sirona) at the WL. All root canal treatments were completed in a single appointment under local anesthesia, with the use of a rubber dam and magnified visualization by a single operator (G.A.P) with 6 years of clinical experience in utilizing reciprocating systems.

### Statistical analyses

The recorded information encompassed details such as tooth type, patient gender and age, reciprocating system employed, number of fractured instruments, and the length of fractured segments. Periapical radiographs were analyzed to ascertain the location of fragments in the root canals. Statistical analyses utilized the chi-square test of goodness of fit, enabling a comparison between observed frequencies of fractures during the second and third instrument uses, and their respective expected frequencies, determined based on fracture incidence during the initial use. The significance level was set at 5% (SPSS v. 22 for Windows; IBM SPSS Statistics Chicago, IL, USA).

## Results

**Figure 1:**
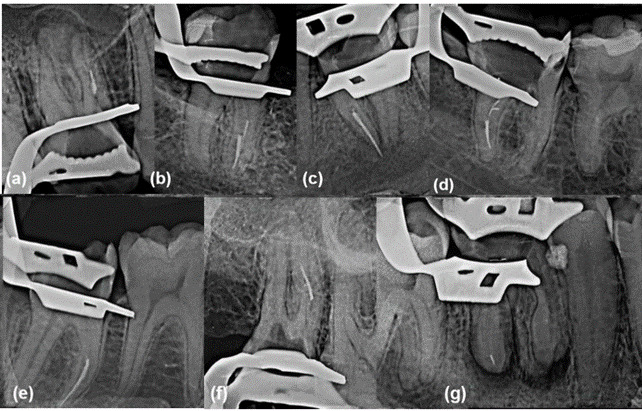
There were 3 fractures over the course of WaveOne Gold usage, with the first occurring in (A) the middle third of the mesiobuccal root of a maxillary first molar (tooth 16), (B) the second in the apical third of the mesial root of a mandibular first molar (tooth 46), (C) and the third during the apical preparation of the mesial root of a mandibular second molar (tooth 37); fragments of 2 Reciproc Blue instruments were found in the apical third of the (D) distal and (E) mesial roots of 2 mandibular second molars (tooth 47); two Reciproc instruments fractured (F) in the apical third of a mesiobuccal canal in a maxillary second molar (tooth 17) and (G) in the apical third of the distal root of a mandibular first molar (tooth 46).

The overall incidence of instrument fracture was 0.21% after the second use and 0.43% after the third use (Table 3). No signs of deformation were observed after each use of the instruments. During the first use, only 1 WaveOne Gold Primary instrument has fractured (0.32%). The results of the statistical tests comparing observed fracture frequencies to expected frequencies after each reuse were as follows: (i) in the Reciproc group, there were no fractures on the second use (*P* = 0.99, *X* ^2^ = 0.000), but 2 fractures on the third use (*P* = 0.000, *X* ^2^ = 124.249); (ii) in the Reciproc Blue group, there was 1 fracture on both second (*P* = 0.000, *X* ^2^ = 30.085) and third (*P* = 0.000, *X* ^2^ = 30.396) uses; (iii) in the WaveOne Gold group, there was 1 fracture on both second (*P* = 0.99, *X* ^2^ = 0.000) and third (*P* = 0.98, *X* ^2^ = 0.000) uses. Fractures occurred in different levels of maxillary and mandibular molar roots, and the length of fractured segments ranged from 1 to 6 mm (Figure 1; Table 3).

**Table 3:** Number of fractured instruments, number of treated canals, percentage frequency of file fracture per use and the affected teeth during the first, second and third uses of Reciproc (RC), Reciproc Blue (RB) and WaveOne Gold (WG) instruments.

	1^st^ use			2^nd^ use			3^rd^ use			Total		
	RC	RB	WG	RC	RB	WG	RC	RB	WO	RC	RB	WG
Number of instruments	0	0	1	0	1	1	2	1	1	2	2	3
Number of canals	312	312	312	312	312	309	312	309	306	936	933	927
% frequency	0	0	0.32	0	0.32*	0.32	0.64*	0.32	0.33	0.21	0.21	0.32
Affected teeth	-	-	46	-	47	16	17,46	47	37	17,46	47,47	16,37,46
Total	1 fractured instrument (0.32%) in 936 canals			2 fractured instruments (0.21%) in 933 canals			4 fractured instruments (0.43%) in 927 canals			7 fractured instruments (0.25%) in 2796 canals		

* Significant statistical difference (*P* < 0.05) between the observed frequencies of fractures at each reuse compared to the respective first use (expected frequency).

## Discussion

Fracture of NiTi instruments is influenced by factors such as the operator's expertise, instrument design, manufacturing process, instrumentation technique, canal configuration, and often result from torsional overload when the instrument's tip binds in the canal while the motor continues to rotate ^19^. Reciprocating kinematics, characterized by alternating rotations, reduces tensional stress on the instrument, preventing torsional failure, and is therefore considered a strategy to minimize instrument fracture ^13^. Despite manufacturers recommending discarding these instruments after the first use due to potential damage accumulation and exceeding elastic limits, a study suggests minimal changes after multiple uses ^11^, prompting further research on the feasibility of reuse, particularly in cost-constrained settings. Another approach to reduce the risk of instrument fracture involves creating a glide path, which is a smooth and continuous pathway established before enlarging the root canal ^4^ ^,^ ^22^ ^,^ ^23^. This procedure typically involves manual instrumentation techniques and is considered completed when either a size 10 or 15 hand file is loose in the canal ^4^. While studies have demonstrated that enlarging the canal to this size reduces the risk of NiTi instrument fracture ^4^ ^,^ ^22^ ^,^ ^23^, it is also possible that a larger pre-enlargement could result in a lower incidence of fracture during instrument reuse. Based on these premises, this clinical study presents original data by evaluating the fracture incidence of Reciproc R25, Reciproc Blue R25 and WaveOne Gold Primary instruments in the preparation of 2,796 root canals of molar teeth (Table 1) when a pre-enlargement using a size 25 K-file was performed before reusing the instruments. Overall, the incidence of instrument fracture was 0.21% after the second use and 0.43% after the third use, indicating potential benefits from using a pre-enlargement with a size 25 K-file in the reuse of reciprocating instruments. Therefore, the null hypothesis was accepted.

The fracture rate of the WaveOne M-Wire instrument has been assessed in some prospective clinical studies, and collectively, these studies have reported fracture rates lower than 0.85% for both single use ^14^ ^,^ ^15^ ^,^ ^16^ and multiple uses ^11^. The lowest incidence (0.13%) was reported by Cunha et al. ^16^ after the preparation of 2,215 canals, which could be explained by the use of Gates-Glidden drills to prepare canal orifices, a procedure that might have contributed to reduce the incidence of fracture. In the only prospective clinical study that has evaluated the fracture rate of the WaveOne Gold instruments published so far, the authors reported no fractures after the single-use preparation of molars ^24^. This finding contrasts with the present outcome, where 1 WaveOne Gold instrument (0.32%) fractured during its initial use and subsequent reuses (Table 3). The variation in results can be partially attributed to the difference in sample size between the two studies. Specifically, the study in question (n = 750 molars) ^24^ had a sample size that was three times smaller than the sample size of the present study. Moreover, a smaller instrument (21/07) was employed in that study ^24^ when the WaveOne Gold Primary instrument (25/07) encountered difficulty advancing in the thirds, a procedure not applied in this study.

In the only study investigating the reuse of Reciproc R25, the authors used 60 instruments for the treatment of 180 teeth (1 instrument per 3 teeth) or 540 canals ^11^. Two Reciproc R25 instruments fractured, with one incident after the initial use and the second after the third use, resulting in a total failure rate of 0.37%. This outcome is consistent with the present finding, where 2 Reciproc R25 instruments fractured (0.21%) after the second and third uses, respectively (Table 3). The fracture incidence of the single use of Reciproc R25 M-Wire has been investigated in other clinical studies as well ^9^ ^,^ ^14^ ^,^ ^17^ ^,^ ^18^. Plotino et al. ^17^ reported that 5 Reciproc R25 instruments fractured in primary treatment cases, accounting for 0.13% of the root canals treated. Caballero-Flores et al. ^14^ evaluated the fracture incidence of the Reciproc R25 instrument when used by dental students and reported a fracture rate of 0.93% during the preparation of 1,126 root canals. In a study conducted by Zuolo et al. ^18^ to evaluate the Reciproc R25 instrument's capability to reach the full WL in the second mesiobuccal canals (MB2) of maxillary molars without a prior glide path, out of 174 MB2 canals, 3 fractured instruments were recorded (1.72%). Using a similar methodology, Rodrigues et al. ^9^ reported 3 instrument fractures (0.44%) during the preparation of 673 MB2 canals. It is important to note that the higher fracture rates observed in some of these studies, where the single use of the Reciproc R25 was employed, compared to the present findings, where the Reciproc R25 was reused, are attributed to more challenging anatomical conditions (MB2 canal) ^9^ ^,^ ^18^ or preparation being conducted by less experienced clinicians ^14^. The fracture incidence of Reciproc Blue R25, not previously studied clinically, is demonstrated for the first time in this prospective clinical study, revealing a low fracture rate comparable to the Reciproc R25 M-Wire instrument (0.21%) (Table 3).

The highest incidence of instrument fractures in this study was found in the apical third of the mesiobuccal root of maxillary molars and the mesial root of mandibular molars (Figure 1). This result is consistent with other studies ^11^ ^,^ ^14^ ^,^ ^16^ and can be attributed to the complex anatomy of these root canals, which often present multiplanar curvatures ^19^ that can be difficult to detect radiographically, especially in the apical third ^25^. The size of the fracture fragments varied from 1 to 6 mm, a finding that is also in line with other publications ^14^ ^,^ ^15^.

In this study, the fracture incidence during repeated use of reciprocating instruments was assessed by comparing it with the fracture rate observed in their initial use, utilizing the chi-square test of goodness of fit. The statistical analysis aimed to determine if the pre-enlargement with a size 25 K-file created in subsequent uses resulted in a fracture rate comparable to the first use. A p-value greater than 0.05 indicated the beneficial effect of this pre-enlargement for a specific file reuse. The WaveOne Gold instrument consistently displayed a comparable fracture rate in the first, second, and third uses, suggesting the efficacy of a pre-enlargement with a size 25 K-file in both reuses. In contrast, the Reciproc Blue instrument did not show the same benefit from this pre-enlargement, with both the second and third uses exhibiting a higher incidence of fracture compared to the first use. The Reciproc M-Wire showed intermediate results, where the pre-enlargement with a size 25 K-file was beneficial only in the second use but not in the third (Table 3).

Despite the strengths of this study, several limitations should be acknowledged. First, the use of stainless-steel K-files for pre-enlargement represents a potential limitation, as these instruments are known to cause deviations and alterations in the original root canal anatomy, especially in larger canals. NiTi K-files could have been employed to minimize the risk of procedural errors. However, it is important to emphasize that the current study includes only canals with curvatures of less than 20º, where the risk of procedural deviations is inherently lower. This choice allowed for consistent instrumentation within the study’s parameters, although the potential for improved outcomes with NiTi K-files in more complex anatomies remains noteworthy. Moreover, the topographic characteristics of the fractured instruments could be evaluated with scanning electron microscopy, which could have provided a more detailed analysis of the surface features and potential causes of instrument failure. Additionally, the study was conducted in a single clinical practice with a specific operator, which may limit the generalizability of the findings. The operator’s expertise and the standardized environment might not fully represent the variability encountered in different clinical settings with operators of varying experience levels. Finally, the study's exclusion criteria, particularly the selection of canals with less than 20º curvature and the exclusion of additional or accessory canals may limit the applicability of the results to more challenging endodontic cases. Future studies should consider including a broader range of anatomical variations to better assess the performance of these instruments in diverse clinical scenarios.

Clinical studies on patients undergoing non-surgical root canal treatment have reported low fracture rates, indicating the potential for reusing reciprocating instruments ^9^ ^,^ ^11^ ^,^ ^14^ ^,^ ^15^ ^,^ ^16^ ^,^ ^17^ ^,^ ^18^ ^,^ ^24^. While the pre-enlargement with a size 25 K-file did not entirely eliminate the risk of fracture after reusing reciprocating instruments, our findings showed fracture rates comparable to other clinical studies that tested their single use with ^11^ ^,^ ^14^ ^,^ ^15^ ^,^ ^16^ ^,^ ^24^ or without ^9^ ^,^ ^17^ ^,^ ^18^ glide path. In the current study, following a pre-enlargement with a size 25 K-file in 1,860 canals, only 6 instruments fractured (0.32%), suggesting that this approach may prolong the lifespan of these instruments and serve as an alternative for their reuse with a reduced risk of fracture. However, a comprehensive comparison of reusing reciprocating instruments with and without a pre-enlargement warrants additional studies. Further clinical research with a larger sample size and consideration of factors such as operator experience, and instrument usage beyond three clinical cases would be beneficial for a more comprehensive understanding of the phenomenon of instrument fracture in clinics.
